# Glycyrrhizic Acid-Induced Differentiation Repressed Stemness in Hepatocellular Carcinoma by Targeting c-Jun N-Terminal Kinase 1

**DOI:** 10.3389/fonc.2019.01431

**Published:** 2020-01-09

**Authors:** Shijiao Cai, Zhun Bi, Yunpeng Bai, Heng Zhang, Denghui Zhai, Cui Xiao, Yuanhao Tang, Lan Yang, Xiaoyun Zhang, Kun Li, Ru Yang, Yanrong Liu, Shuang Chen, Tao Sun, Huijuan Liu, Cheng Yang

**Affiliations:** ^1^State Key Laboratory of Medicinal Chemical Biology and College of Pharmacy, Nankai University, Tianjin, China; ^2^Tianjin Key Laboratory of Molecular Drug Research, Tianjin International Joint Academy of Biomedicine, Tianjin, China; ^3^College of Life Sciences, Nankai University, Tianjin, China

**Keywords:** glycyrrhizic acid, stemness, differentiation, JNK1, sorafenib

## Abstract

Hepatocellular carcinoma (HCC) is one of the most common malignant cancers with poor prognosis and high incidence. Cancer stem cells play a vital role in tumor initiation and malignancy. The degree of differentiation of HCC is closely related to its stemness. Glycyrrhizic acid (GA) plays a critical role in inhibiting the degree of malignancy of HCC. At present, the effect of GA on the differentiation and stemness of HCC has not been reported, and its pharmacological mechanism remains to be elucidated. This study evaluated the effect of GA on the stemness of HCC and investigated its targets through proteomics and chemical biology. Results showed that GA can repress stemness and induce differentiation in HCC *in vitro*. GEO analysis revealed that cell differentiation and stem cell pluripotency were up-regulated and down-regulated after GA administration, respectively. Virtual screening was used to predict the c-Jun N-terminal kinase 1 (JNK1) as a direct target of GA. Moreover, chemical biology was used to verify the interaction of JNK1 and GA. Experimental data further indicated that JNK1 inhibits stemness and induces differentiation of HCC. GA exerts its function by targeting JNK1. Clinical data analysis from The Cancer Genome Atlas also revealed that JNK1 can aggravate the degree of malignancy of HCC. The results indicated that, by targeting JNK1, GA can inhibit tumor growth through inducing differentiation and repressing stemness. Furthermore, GA enhanced the anti-tumor effects of sorafenib in HCC treatment. These results broadened our insight into the pharmacological mechanism of GA and the importance of JNK1 as a therapeutic target for HCC treatment.

## Introduction

Hepatocellular carcinoma (HCC) is one of the most common malignant cancers worldwide (1). Cancer stem cells (CSCs) play a vital role in tumor progression because of their self-renewal and infinite proliferation properties (2). In experimental models, the existence of CSCs is one of the major factors causing resistance to conventional radiotherapy and chemotherapy (3–5). HCC exhibits poor differentiation and unlimited proliferative capacity. Mutations occurring in well-differentiated cells can lead to increase numbers of self-renewing cells (6, 7).

HCC dedifferentiation contributes to malignant progression, which is characterized by a significant change of morphology and loss of hepatic function (8). The induction of HCC differentiation is regarded as a prospective strategy for HCC treatment (9–11). Numerous studies have elucidated that poor differentiation can lead to high recurrence rates (12, 13). Differentiation markers, such as the fetal liver marker AFP, is highly expressed in poorly differentiated HCCs, whereas the hepatocyte lineage differentiation marker HEPPAR1 presents low expression (13–15). Evaluating the differentiation degree of HCC is vital to the development of therapeutic strategies. These findings suggest that differentiation therapy may be an effective method to treat HCC.

Glycyrrhizic acid (GA) is the main active ingredient in *Glycyrrhiza uralensis Fisch*, a commonly used traditional Chinese medicine (TCM). GA is widely used as a therapeutic agent to cure chronic liver diseases. In addition, it can exert an anti-tumor effect by repressing angiogenesis (16) and inhibiting metastasis (17). Thus, far, the effect of GA on the differentiation and stemness of HCC has not been reported, and its pharmacological mechanism remains to be elucidated.

c-Jun N-terminal kinase 1 (JNK1), a member of the JNK family, plays a vital role in malignant transformation. JNK1 is involved in regulating CSC, and high JNK1 activation is closely associated with poor prognosis in HCC patients (18, 19). Blocking the JNK1 signaling cascade with its inhibitor SP600125 can reduce the CSC population and enhance differentiation in glioma (20). A previous study has demonstrated that the JNK1 pathway can sufficiently block the differentiation of leukemia cells (21). Another prior study revealed that differentiation is obviously down-regulated in HCC samples with high JNK1 activation (22). Therefore, strategies to inhibit the levels and activities of JNK1 may be effective for HCC prevention and therapy.

However, the potential target of GA and its mechanisms remain unclear. This study was the first to evaluate the effect of GA on the differentiation and stemness in HCC. We clarified that GA can inhibit tumor growth by inducing differentiation and repressing stemness. Moreover, JNK1 was found to be the direct target of GA. JNK1-knockdown-medicated differentiation likewise dramatically inhibits stemness. In conclusion, GA-induced differentiation represses stemness in HCC by targeting JNK1. The results of our study may be used to develop more efficient guidelines to treat HCC.

## Materials and Methods

### Cell Culture

The HCC cell lines (HepG2 and PLC/PRF/5) were purchased from KeyGen Biotech (Nanjing, China) and cultured in Dulbecco's modified Eagle's medium and RPMI 1640 medium, respectively. The complete medium was supplemented with 10% fetal bovine serum and 1% penicillin-streptomycin. The cells were maintained at 37°C in a humidified atmosphere including 5% CO_2_.

### Cell Viability Assay

Cell viability was assessed by using the MTT method. Cells were seeded in a 96-well plate at a density of 1 × 10^4^ cells/well. Different concentrations of GA (0, 1, 2, 3, 4, and 5 mM, Meilunbao, Dalian, China) were added after 24 h. After 48 h of continuous exposure to GA, 10 μL of MTT (5 mg/mL) was added and incubated for another 4 h at 37°C. Afterwards, 100 μL of dimethyl sulfoxide (DMSO) was added to dissolve formazan crystals. Cell viability was determined by measuring the optical density at 570 nm with a microplate reader (Multiskan™ FC, Thermo Scientific, Waltham, MA, USA). The experiments above were performed in triplicate.

### Clone Formation Assay

The cells were seeded in 6-well plate at a density of 400 cells per well. For pharmacodynamic test, the cells were continuously maintained in different concentrations of GA (0, 0.5, 1, and 2 mM). For target validation experiment, PLC/PRF/5 cells transfected with siNC or siJNK1 were continuously exposed to 0 or 2 mM GA. Cells were incubated for ~10 days to form sizeable colonies. The colonies were fixed with 4% paraformaldehyde for 20 min at room temperature and then stained with 0.1% crystal violet to visualize the colonies. The experiments above were performed in triplicate. Photographs were taken by a microscope (Nikon, Japan).

### Sphere Formation Assay

The cells were collected and rinsed to remove serum and then dissociated to a single-cell suspension in 3D Tumor Sphere Medium XF (Promocell, Sickingenstr, Heidelberg, Germany). For pharmacodynamic test, cells were continuously incubated with medium containing different concentrations of GA (0, 0.5, 1, and 2 mM). For target validation, PLC/PRF/5 cells transfected with siNC or siJNK1 were continuously exposed to medium containing 0 or 2 mM GA. Cells were subsequently cultured in an ultra-low attachment 24-well plate at a density of 1,000 cells per well for ~10 days. Images were photographed using a microscope (Nikon, Japan). The experiments above were performed in triplicate.

### Western Blot Assay

Proteins were extracted with a RIPA buffer containing phenylmethane sulfonyl fluoride (Sigma, St. Louis, MO, USA) and a protease inhibitor cocktail (Sigma). Then, 20 μg of protein was separated by 8–12% Tris-acrylamide gels and incubated with primary antibodies overnight at 4°C. Afterwards, the membranes were incubated with horseradish peroxidase (HRP)-conjugated secondary antibodies for 1 h at room temperature. The primary antibodies used were as follows: anti-AFP (1:500; Affinity), anti-GAPDH (1:1,000; Affinity, Cincinnati, OH, USA), anti-HEPPAR1 (1:500; Novus Biologicals Centennial, CO, USA), anti-JNK1 (1:1,000; Abcam, Cambridge, UK), anti-OCT4 (1:1,000; Abcam), and anti-SOX2 (1:1,000; Abcam). The results were detected through an ECL reagent (Millipore, Billerica, MA, USA) and captured by an electrophoresis gel imaging system (ChemiScope 6000, CLIX, Shanghai, China). The experiments above were performed in triplicate.

### Bioinformatics Analysis

Data related to HepG2 treated with GA were downloaded from GEO databases. The GEO series accession number is GSE67504. Significantly different expression was identified as up-regulated or down-regulated according to the following standard: ANOVA *P* < 0.05, |Fold change| > 1.5. Hierarchical clustering was generated by the R package (pheatmap). The functions of down-regulated and up-regulated genes were analyzed with Metascape and visualized in Cytoscape. Data related to JNK1 in HCC were acquired from The Cancer Genome Atlas (TCGA). Patient samples were classified into either JNK1-high or JNK1-low group. Gene Set Enrichment Analysis (GSEA) analysis was performed on the basis of JNK1 mRNA expression (23, 24).

### Synthesis of GA Probe

Synthesis of GA-yne was performed using the purchased GA as the raw material. The terminal alkyne-containing GA-yne probe was synthetized by linking GA to 2-(3-but-3-ynyl-3H-diazirin-3-yl)-ethanol. The fluorescent group rhodamine-N3 was synthesized in accordance with previously published procedures (25).

### Molecular Docking

Molecular docking was performed using Sybyl X1.1 software. The crystal structure of JNK1 was downloaded from the PDB database (PDB code, 2XS0). The 3-D structure of the GA was formed with LigPrep. Docking score was used to screen out the potential target of GA amongst multiple proteins.

### Immunofluorescence Assay

GA-yne was added when HepG2 cells seeded in dishes had grown to 70% confluence. After 5 h, UV irradiation (350 nm) was performed for 30 min. The cells were fixed with 4% formaldehyde and then blocked with 5% FBS, including 0.1% TritonX-100. Afterwards, a solution (1 mM/L CuSO_4_, 1 mM/L TCEP, 100 μM/L rhodamine-N_3_, and 100 μM/L TBTA dissolved in PBS) was added to generate click chemistry reaction. Samples were incubated overnight with primary antibody JNK1 (1:100; Abcam) at 4°C and then with secondary antibodies combined with Alexa Fluor 488 (Invitrogen, Waltham, MA, USA) for 1 h at room temperature. The images were obtained with a confocal microscope (Nikon, Japan).

### Super-Resolution Microscopy

HepG2 cells were seeded in 35 mm dishes (World Precision Instruments, USA) and then grown to 60% confluency. Afterwards, a GA probe was added and incubated for 4 h followed by UV irradiation (350 nm) for 30 min. The GA probe was coupled with 647-conjugated azide (Thermo Fisher, USA) by click chemistry reaction after cells were fixed and blocked. Subsequently, cells were incubated overnight with primary antibody JNK1 (1:100; Abcam) at 4°C and then with Cy3B-conjugated goat anti-rabbit secondary antibodies for 1 h at room temperature. Images were captured with a Nikon stochastic optical reconstruction microscope (N-STORM, Nikon, Japan).

### Biacore Assay

Biacore assay was carried out using a Biacore 3000 instrument (GE Healthcare, Piscataway, NJ, USA). JNK1 was coupled to CM5 sensor chips activated by 50 mM NHS and 200 mM EDC (at a ratio of 1:1). Afterwards, GA was diluted in a buffer and then injected into JNK1-immobilized CM5 sensor chips at concentrations of 3.125, 6.25, 15.625, 31.25, and 62.5 μM. All signals were adjusted by a reference channel. Results were analyzed by using the BIA evaluation software.

### RNA Interference

All siRNAs were transfected using Lipofectamine RNAi MAX following the standard protocol. The PLC/PRF/5 and HepG2 cells were collected after 72 h of the experiments. Negative control siRNA sequence: 5′- UUCUCCGAACGUGUCACGUTT-3′. JNK1 siRNA: 5′- GCUGGUAAUAGAUGCAUCUTT-3′.

### Lentiviral Production

The sequences of shRNA used in this study are as follows. shNC: CCGGTTTCTCCGAACGTGTCACGTTTCAAGAGAACGTGACACGTTCGGAGAATTTTTTG; shJNK1: CCGGTGTGTCTTCAATGTCAACAGCTTCCTGT CAGACTGTTGACATTGAAGACACTTTTTTG. The palindromic DNA oligo was annealed to form a double-strand oligo and then ligated to the linearized pLKD-CMV-EGFP-2A-Puro-U6-shRNA (OBIO, Shanghai) vector to generate circled pLKD-CMV-shRNA-Puro. pLKD-CMV-shRNA-Puro, pLP1, pLP2, and VSV-G were then co-transfected into HEK 293T cells by using Lipofectamine 2000 (Invitrogen, Grand Island, NY, USA). PLC/PRF/5 cells were infected with lentivirus carrying pLKD-CMV-shJNK1-puro or pLKD-CMV-shNC-puro plasmids, followed by selection using 2 mg/mL puromycin to generate stablely transfected cell lines.

### Tumor Xenograft

Four- to five-week-old female BALB/c nu/nu mice were raised in specific pathogen-free (SPF) conditions at Tianjin International Joint Academy of Biomedicine. For target validation experiment, PLC/PRF/5 cells stably transfected with shNC or shJNK1 were injected subcutaneously into nude mice (2 × 10^6^ cells in 100 μL PBS), which were then randomly divided into four groups (*n* = 4). When the tumor volume reached ~50 mm^3^, the mice were treated by gavage with 100 mg/kg GA daily or with saline as control. For combination experiment, PLC/PRF/5 cells were injected subcutaneously into nude mice (2 × 10^6^ cells in 100 μL PBS), which were then randomly divided into four groups (*n* = 4). The mice in the experiment groups were treated by gavage with GA (100 mg/kg daily), sorafenib (10 mg/kg daily) or a combination of GA (100 mg/kg daily), and sorafenib (10 mg/kg daily) when the tumor volume reached ~50 mm^3^. Meanwhile, the mice in the control group were treated with the same volume of saline. Body weight and tumor diameter were measured every 3 days. Tumor volumes were evaluated using the following formula: *V* = length × width^2^/2 (26). Finally, all mice were euthanized simultaneously and the tumors were subjected to immunohistochemistry (IHC) staining. All animal experiments were performed under the approved protocols of the Institutional Animal Care and Use Committee.

### Immunohistochemistry

The tumors were fixed in 10% formalin and embedded in paraffin followed by serial transverse sections (5 μm). The sections were deparaffinized, dehydrated, and rehydrated before IHC was performed. After blocking with 10% normal goat serum for 20 min, the sections were incubated overnight with primary antibodies at 4°C. The primary antibodies were listed as follows: anti-AFP (1:100; Affinity), anti-HEPPAR1 (1:200; Novus Biologicals), anti-JNK1 (1:100; Abcam), anti-OCT4 (1:100; Abcam), and anti-SOX2 (1:100; Abcam). Subsequently, HRP-conjugated secondary antibodies were dropped into the sections for 1 h at room temperature. The sections were then stained with the 3,3-diaminobenzidine (DAB) solution and counterstained with hematoxylin. Photographs were captured with an Olympus light microscope (Nikon, Japan).

### Statistical Analysis

Statistical analysis was conducted using GraphPad software (version 7, GraphPad Software, Inc., La Jolla, CA, USA). Data were presented as means ± SD. One-way ANOVA was used to compare the multiple groups of data. Survival curve was analyzed using Kaplan–Meier method with logrank (Mantel-Cox test). *P* < 0.05 was considered statistically significant.

## Results

### GA Reduces Stemness and Induces the Differentiation of Hepatic Cancer Cells

**Figure 1:**
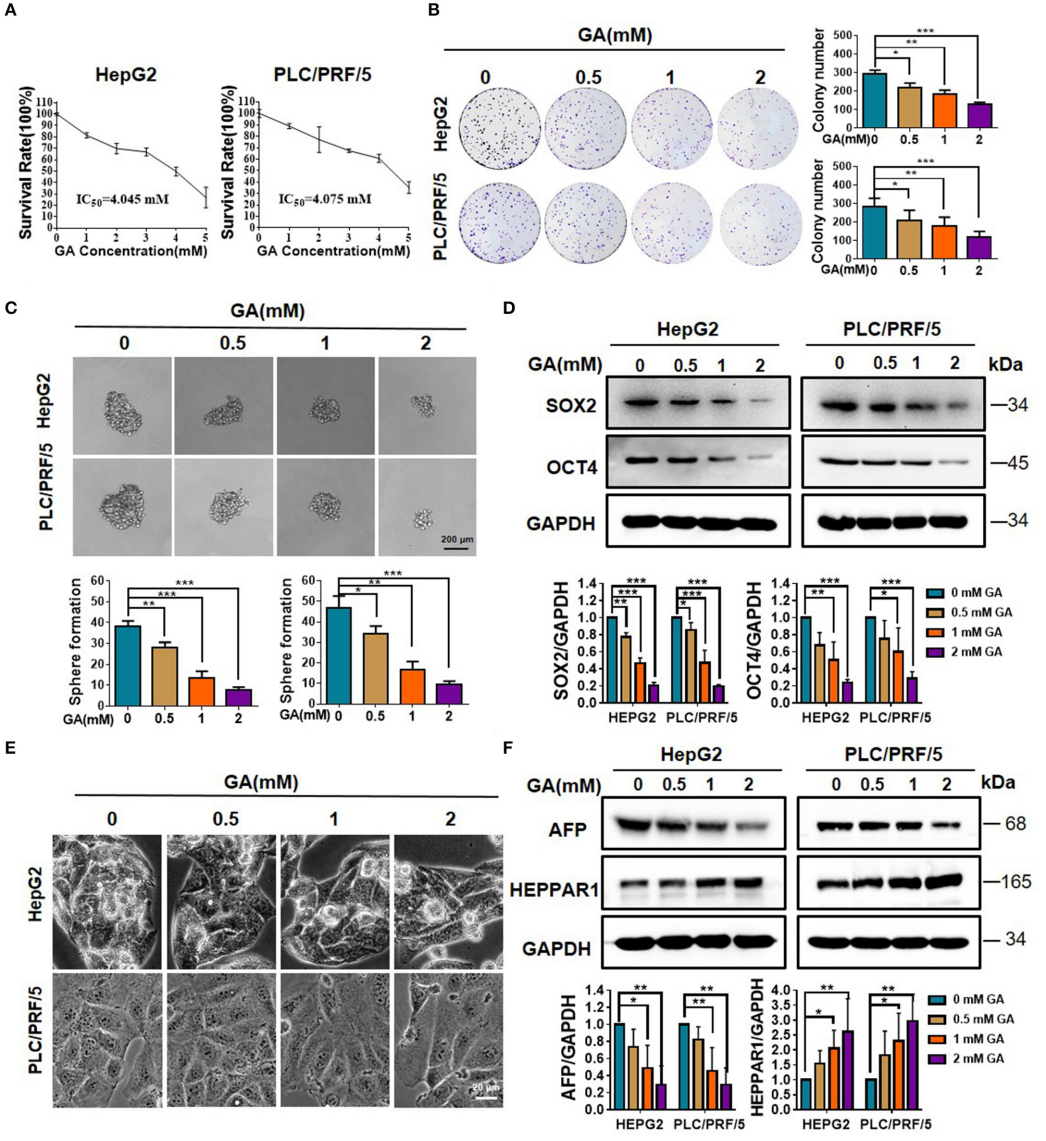
GA reduces stemness and induces differentiation of hepatic cancer cells. **(A)** Survival of HepG2 and PLC/PRF/5 cells incubated with the indicated amounts of GA for 48 h. **(B)** Cell proliferation was assessed through a colony formation assay. The number of colonies was compared (one-way ANOVA; ^*^P < 0.05, ^**^P < 0.01, ^***^P < 0.001). **(C)** Images of sphere formation assay of HepG2 and PLC/PRF/5. The number of spheres was compared (one-way ANOVA; ^*^P < 0.05, ^**^P < 0.01, ^***^P < 0.001). **(D)** Expression of representative CSC markers (SOX2 and OCT4) was analyzed by Western blot assay (one-way ANOVA; ^*^P < 0.05, ^**^P < 0.01, ^***^P < 0.001). **(E)** Images of HepG2 and PLC/PRF/5 cells were taken after incubation with different GA concentrations. **(F)** Expressions of representative differentiation markers (AFP and HEPPAR1) were analyzed by Western blot assay (one-way ANOVA; ^*^P < 0.05, ^**^P < 0.01). Scale bars in C and E are at 200 and 20 μm, respectively.

We first performed MTT on HepG2 and PLC/PRF/5 to detect the effect of GA on cell viability. The cells were then incubated with various concentrations of GA (0, 1, 2, 3, 4, and 5 mM) for 48 h. The half-maximal inhibitory concentrations (IC_50_) in HepG2 and PLC/PRF/5 cells were 4.045 and 4.075 mM, respectively (Figure 1A). Afterwards, the effects of GA on proliferation were evaluated via colony formation assay. The results showed that GA can inhibit proliferation in a concentration-dependent manner (Figure 1B; one-way ANOVA, ^*^P < 0.05, ^**^P < 0.01, ^***^P < 0.001). The effects of GA on stemness were then evaluated via sphere formation assay. Results showed that GA can reduce the number and size of the spheres in a dose-dependent manner compared with those of the control group (Figure 1C; one-way ANOVA, ^*^P < 0.05, ^**^P < 0.01, ^***^P < 0.001). The protein levels of stem cell markers, such as SOX2 and OCT4, dramatically decreased in a dose-dependent manner after GA incubation compared with the unexposed group (Figure 1D; one-way ANOVA; ^*^P < 0.05, ^**^P < 0.01, ^***^P < 0.001). To explore whether GA can induce the differentiation of HCC, we observed the HepG2 and PLC/PRF/5 cell phenotype and found that GA can lead to marked morphological changes compared with the control (Figure 1E). In addition, the expression of AFP was significantly decreased, whereas that of HEPPAR1 was dramatically increased in a dose-dependent manner (Figure 1F; one-way ANOVA; ^*^P < 0.05, ^**^P < 0.01). Taken together, these data indicated that GA can reduce stemness and induce differentiation in HepG2 and PLC/PRF/5 cells.

### Multiple Functions in HCC Are Affected After GA Treatment

**Figure 2:**
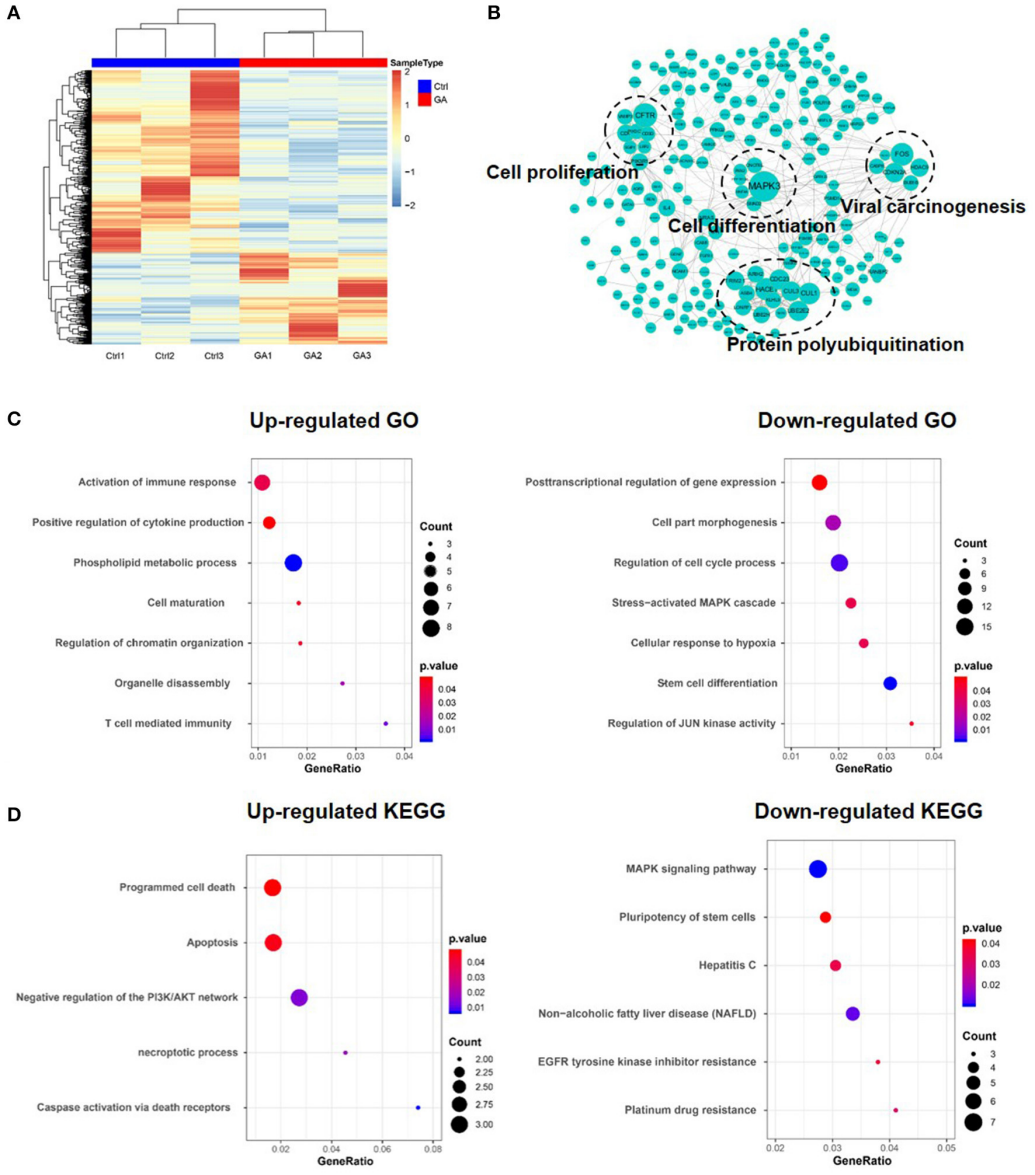
Multiple functions in HCC were affected after treatment with GA. **(A)** Heat map of the differential gene expression profile in HepG2 cells after GA treatment. **(B)** Functions were analyzed using Metascape and shown in Cytoscape. **(C)** GO analysis of up-regulated and down-regulated genes. **(D)** KEGG analysis of up-regulated and down-regulated genes.

Metascape was used to confirm the functional enrichment of multiple genes (27). To investigate the functions affected after GA treatment, we downloaded and analyzed data (HepG2 treated with GA) from the GEO database (GSE67504). The heat map generated from differential genes is shown in Figure 2A. Down-regulated and up-regulated genes are marked in blue and red, respectively. The function of the differential protein was analyzed using the Metascape database and presented in Cytoscape (Figure 2B). Gene ontology (GO) and KEGG analysis of the up-regulated and down-regulated genes revealed that GA can promote cell maturation, inhibit proliferation, reduce the pluripotency of stem cells and decrease EGFR tyrosine kinase inhibitor resistance (Figures 2C,D). These results demonstrated that GA can result in terminal maturation and loss of self-renewal. The results also implied that GA can suppress the proliferation and stemness of HCC cells while inducing their differentiation.

### Synthesis and Target Validation of GA Probe

**Figure 3:**
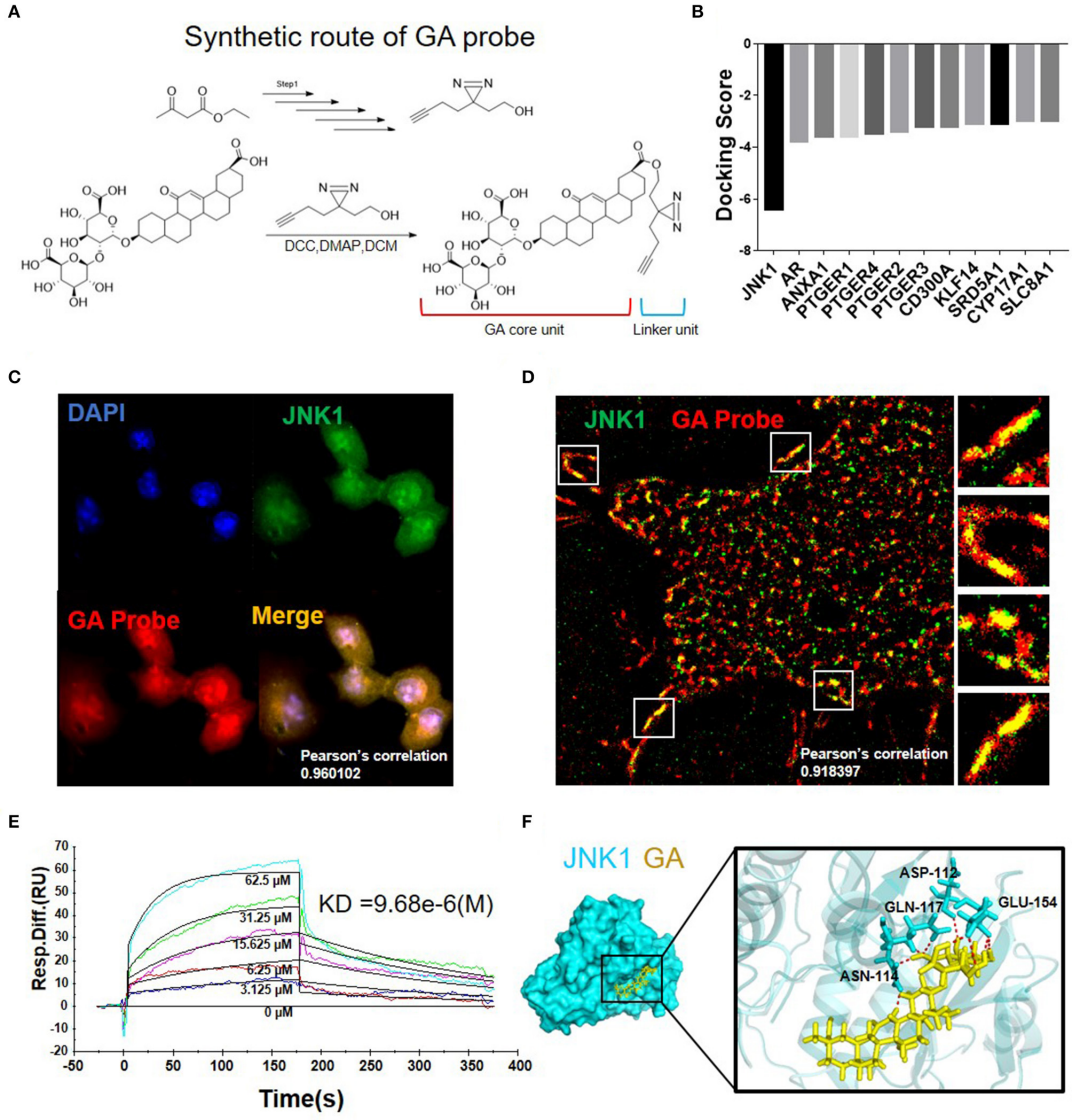
Synthesis and target validation of GA probe. **(A)** Synthetic route and structure of the GA probe used in the study. **(B)** Molecular docking results of GA with multiple proteins. **(C)** Immunofluorescence co-localization of GA probe and JNK1. **(D)** N-STORM picture of GA probe bound with JNK1. **(E)** Biacore analysis revealed that GA can bind well with JNK1. **(F)** Image of molecular dynamics simulation visualizes the combination of GA with JNK1.

The GA probe (GA-yne) was synthesized to confirm the GA target. The synthesis of the GA probe is shown in Figure 3A. The GA probe (GA-yne) was composed of a core unit and a linker unit (28, 29). To screen out the potential target of GA, virtual screening was performed and JNK1 was chosen as the GA target according to the docking score (Figure 3B). First, we performed an immunofluorescence assay in HepG2 and then observed the co-localization of the GA probe and JNK1 under confocal microscopy. The result clearly demonstrated that the GA probe (red) and immunofluorescence of JNK1 (green) co-located, with a Pearson's correlation of 0.960102 (Figure 3C). Furthermore, N-STORM was used to observe the co-localization of the GA probe and JNK1. Figure 3D illustrates that the GA probe and JNK1 are well co-located, with a Pearson's correlation of 0.918397. A Biacore experiment was carried out to further confirm the interaction of GA and JNK1. The dissociation constant (KD) value was 9.68e-6 (M) (Figure 3E). Finally, the molecular dynamics simulation visualized the combination of GA with JNK1, and the binding sites between GA and JNK1 were ASN-114, ASP-112, GLN-117, and GLU-154 (Figure 3F). These results above demonstrated that GA directly targets JNK1 to block the downstream pathway.

### GA Reduces Stemness and Induces Differentiation by Targeting JNK1

**Figure 4:**
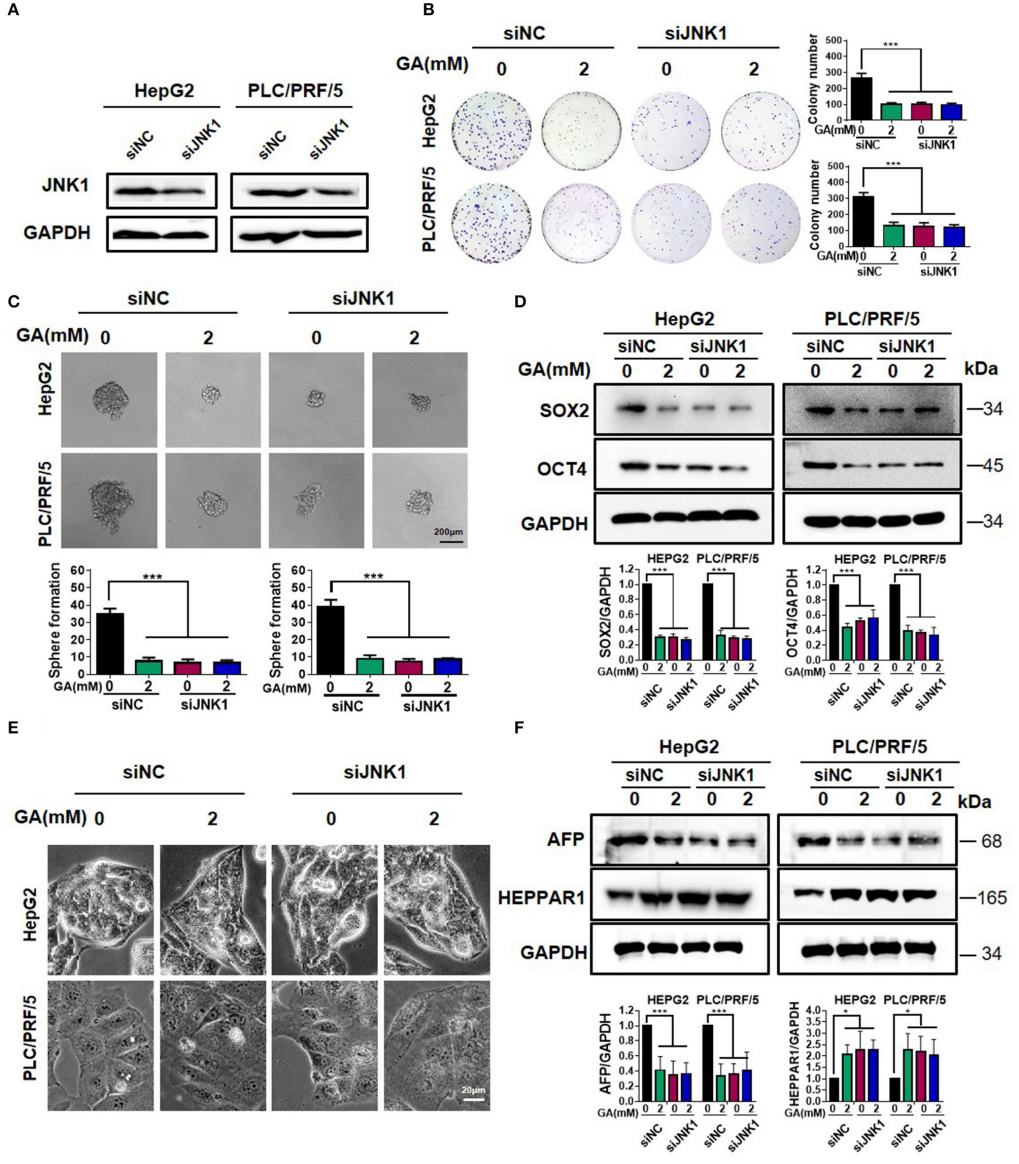
GA reduces stemness and induces differentiation by targeting JNK1. **(A)** JNK1 expression in HepG2 and PLC/PRF/5 cells after being transfected with siNC and siJNK1 plasmids. Colony formation assay **(B)** and sphere formation assay **(C)** in HepG2 and PLC/PRF/5 cells transfected with siNC and siJNK1, followed by treatment with DMSO or GA (2 mM) for 48 h. The numbers of colonies and spheres were compared (one-way ANOVA; ^***^P < 0.001). **(D)**. Expressions of representative CSC markers (SOX2 and OCT4) were analyzed by Western blot assay (one-way ANOVA; ^***^P < 0.01). **(E)** Images of HepG2 and PLC/PRF/5 cells transfected with siNC and siJNK1 followed by incubation with DMSO or GA (2 mM) for 48 h separately. **(F)** Representative markers of differentiation (AFP and HEPPAR1) were analyzed by Western blot assay (one-way ANOVA; ^*^P < 0.05, ^***^P < 0.001). Scale bars in **(C,E)** are at 200 and 20 μm, respectively.

To investigate the mechanism of GA, we knocked down JNK1 and performed Western blot assay. The results clearly showed that JNK1 expression was down-regulated in HepG2 and PLC/PRF/5 cells after being transfected with the siJNK1 plasmid compared with siNC (Figure 4A). JNK1 knockdown and addition of GA alone obviously inhibited colony formation. When JNK1 was knocked down, GA showed no significant inhibitory effect on colony formation compared with DMSO (Figure 4B; one-way ANOVA, ^***^P < 0.001). Meanwhile, either adding GA alone or knocking down JNK1 dramatically reduced sphere formation and expression of CSC markers. Nevertheless, no remarkable difference in the siJNK1 group was observed with or without GA treatment (Figures 4C,D; one-way ANOVA, ^***^P < 0.001). Similarly, knocking down JNK1 or adding GA alone could reverse the poor differentiation of HCC to well-differentiation, and the expression of differentiation markers exerted corresponding changes. However, when JNK1 was knocked down, the degree of differentiation showed no difference in the GA group compared with that in the DMSO group (Figures 4E,F; one-way ANOVA, ^*^P < 0.05, ^***^P < 0.001). In addition, GA blocked the effect on stemness and differentiation induced by JNK1 over-expression (Figure S1). These results further demonstrated that GA represses stemness and induces differentiation in HCC by targeting JNK1.

### JNK1 Can Aggravate the Degree of Malignancy of HCC

**Figure 5:**
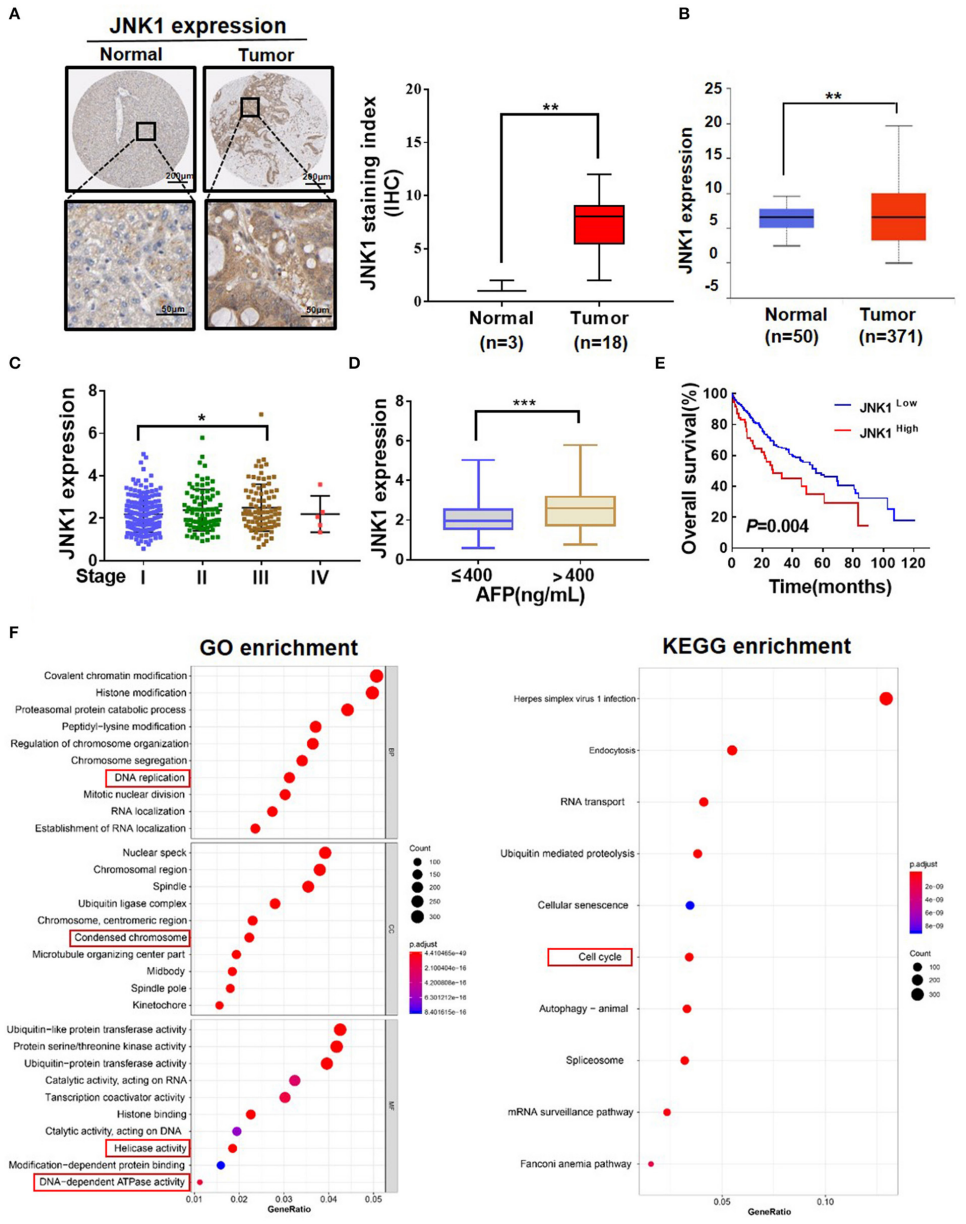
JNK1 can aggravate the degree of malignancy of HCC. **(A)** Representative IHC images of JNK1 in normal tissues and tumors downloaded from the Human Protein Atlas (one-way ANOVA; ^**^P < 0.01). **(B)** Statistical analysis of JNK1 expression level in normal tissues and tumors downloaded from TCGA database (one-way ANOVA; ^**^P < 0.01). **(C,D)** Statistical analysis of JNK1 expression level in liver hepatocellular carcinoma (LIHC) based on clinical stage and AFP level (one-way ANOVA; ^*^P < 0.05, ^***^P < 0.001). **(E)** Survival curve analysis based on low and high JNK1 expressions in LIHC samples. **(F)** GO and KEGG enrichment analysis of JNK1-positive genes.

Clinical data analysis was conducted to investigate the role of JNK1 in HCC. A representative image of IHC downloaded from The Human Protein Atlas database and a statistical analysis illustrated that JNK1 expression is higher in tumors than in normal tissues (Figure 5A; one-way ANOVA, ^**^P < 0.01). The UALCAN database was used to analyze the JNK1 expression. Similarly, JNK1 expression was found to be significantly higher in tumors than in normal tissues (Figure 5B; one-way ANOVA, ^**^P < 0.01). JNK1 expression was positively correlated with the clinical stage and AFP level in TCGA (Figures 5C,D; one-way ANOVA, ^*^P < 0.05, ^***^P < 0.001). Subsequently, we performed survival analysis, and the results indicated that high JNK1 expression predicts poor prognosis (Figure 5E). GO and KEGG enrichment analysis likewise implied that JNK1 can accelerate proliferation (Figure 5F). GO enrichment consisted of biological process (BP), cellular component (CC), and molecular function (MF). As shown in Figure 5F, DNA replication in BP, condensed chromosome in CC and helicase activity and DNA-dependent ATPase activity in MF were closely associated with proliferation. In KEGG enrichment, the cell cycle was closely correlated to proliferation. Functions related to proliferation are highlighted in red boxes. Taken together, these data revealed that JNK1 can aggravate the degree of malignancy of HCC.

### GA Suppresses Tumor Growth and Enhances the Anti-tumor Effect of Sorafenib

**Figure 6:**
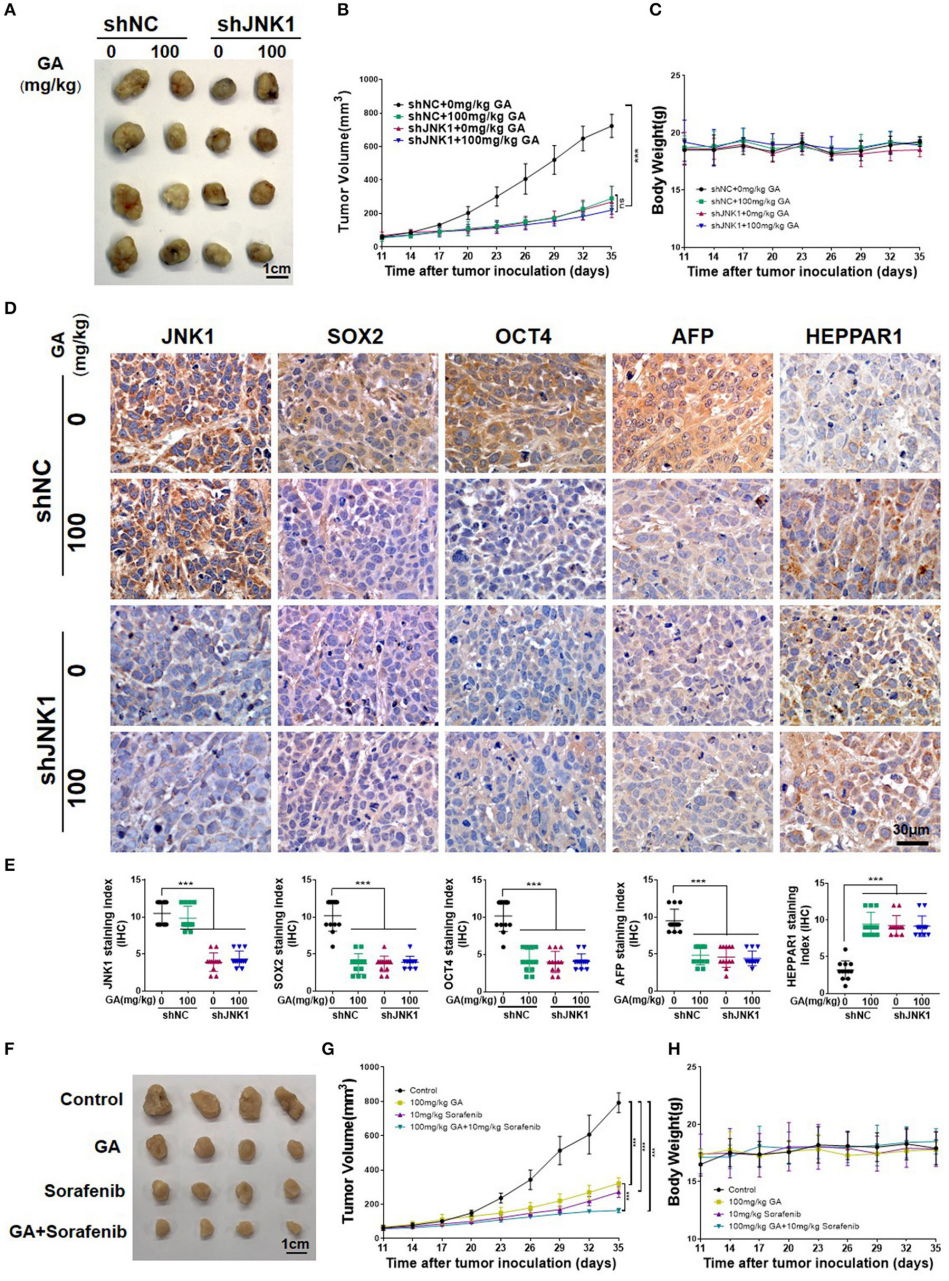
GA suppresses tumor growth and enhances the antitumor effect of sorafenib. Images of tumors **(A)**, tumor volumes **(B)**, and body weight **(C)** of PLC/PRF/5 transfected with shNC or shJNK1 in BALB/c nu/nu mice treated with 100 mg/kg GA or saline as control. **(D)** IHC staining indicates the expression of CSC markers (SOX2 and OCT4) and differentiation markers (AFP and HEPPAR1) in tumors. **(E)** IHC analysis of SOX2, OCT4, AFP, and HEPPAR1 in tumors (one-way ANOVA; ^***^P < 0.001). Scale bars in **(D)**, 30 μm. Images of tumors **(F)**, tumor volumes **(G)** and body weight **(H)** of PLC/PRF/5-bearing BALB/c nu/nu mice. GA or sorafenib treatment obviously inhibited tumor growth. Furthermore, GA could enhance the anti-tumor effect of sorafenib.

To assess the anti-tumor effect of GA, we subcutaneously injected BALB/c nu/nu mice with PLC/PRF/5 transfected with shNC or shJNK1. Tumors images, tumor weight (Figure S2) and tumor volumes indicated that the degree of tumor malignancy was significantly decreased in the shJNK1 group or when 100 mg/kg GA alone was administered compared with the shNC group. Nevertheless, JNK1 knockdown cells exhibited no response to GA (Figures 6A,B; one-way ANOVA, ^***^P < 0.001). These results demonstrated that the anti-tumor effect of GA is JNK1 dependent. Moreover, GA exhibited no influence on the body weight of shNC or shJNK1 group compared with the control (Figure 6C).

The IHC analysis of SOX2 and OCT4 also indicated the stemness is inhibited after GA treatment or JNK1 knockdown. Meanwhile, AFP and HEPPAR1 expression dramatically decreased and increased, respectively, after GA was administered or when JNK1 was knocked down, transforming poorly differentiated HCC to well-differentiated HCC. Nevertheless, no remarkable difference in stemness and differentiation was observed in the shJNK1 group regardless of GA addition (Figures 6D,E; one-way ANOVA, ^***^P < 0.001). These results indicated that GA reduces stemness and induces differentiation by targeting JNK1 *in vivo*.

Sorafenib, is a multikinase inhibitor that has shown efficacy against a wide variety of tumors in preclinical models (30). It can exert antitumor effect through blocking cell proliferation and angiogenesis. Considering the inhibitory effect of GA on the EGFR tyrosine kinase inhibitor resistance of HCC, we hypothesized that combining GA with sorafenib can enhance the antitumor effect of sorafenib. Combination effects were then evaluated in the PLC/PRF/5-bearing BALB/c nu/nu mice. Tumor volumes were significantly smaller in the GA- or sorafenib-treated groups than in the control group. Moreover, GA enhanced the antitumor effect of sorafenib on tumor growth compared with sorafenib alone (Figures 6F,G; one-way ANOVA, ^***^P < 0.001). Overall, GA could suppress tumor growth and sensitize HCC cells for sorafenib. Moreover, GA exhibited no influence on the body weight of GA, sorafenib or a combination of GA and sorafenib group compared with the control (Figure 6H).

## Discussion

Our study elucidated that GA can reduce stemness and induce differentiation by targeting JNK1 *in vitro* and *in vivo*. Malignant HCC cells undergo unlimited proliferation and feature dedifferentiation (31). Dedifferentiation is a virtual event during tumorigenesis, which features morphological changes. Differentiation induced by the effective ingredient of TCM is a new trend in the development of differentiation therapy to treat tumors. Drugs can induce differentiation in many tumor cells, suggesting their clinically importance (32, 33). Differentiation therapy is a potential method to cure tumors (9–11). GO analysis indicated that the regulation of cell differentiation is dramatically up-regulated after GA treatment. The major strength of our study is that it is the first to report the capability of GA to induce HCC differentiation.

GA can exert an anti-tumor effect by reducing proliferation (34, 35). Consistent with a previous study, GO analysis revealed that regulation of the cell cycle process is dramatically down-regulated after GA treatment. Importantly, no studies have reported the effect of GA on the pluripotency of stem cells in HCC. On the basis of the down-regulated GO, the result indicated that the pluripotency of stem cells is dramatically inhibited after GA treatment. Sorafenib, a multi-kinase inhibitor with efficacy against HCC in clinical application, showed drug resistance upon long-term administration. Hagiwara et al. reported that activated JNK and high CD133 expression contributed to poor response to sorafenib in HCC (36, 37). Interestingly, our study indicated that combination with GA can effectively enhance the anti-tumor effect of sorafenib. This study provides new theoretical guidance to improve the anti-tumor effects of sorafenib by its combination with natural drugs.

High JNK1 expression is closely associated with poor prognosis and increases the degree of malignancy of HCC (18, 19, 38). Similarly, in our study, the data analysis based on TCGA illustrated that JNK1 can aggravate the degree of malignancy of HCC. Activated JNK1 is positively correlated with the maintenance of stemness in gliomas, indicating the potential of JNK1 as a target for eliminating stemness. Furthermore, JNK1 blockage inhibits self-renewal and induces differentiation in gliomas (20). Our finding about the relationship of stemness and differentiation are well-consistent with those reported by Chen et al. (39).

In summary, our data showed for the first time that GA reduces the properties of CSCs and induces the differentiation of HCC via JNK1. Our study suggested that GA in combination with sorafenib may be an effective strategy for HCC therapy in the future.

## Data Availability Statement

Publicly available datasets were analyzed in this study. This data can be found here: https://www.ncbi.nlm.nih.gov/geo/query/acc.cgi?acc=GSE67504.

## Author Contributions

CY, HL, and TS conceived and designed the experiments. SCa, ZB, YB, and DZ performed all of the experiments. CX, YT, LY, KL, XZ, RY, YL, and SCh provided technical and material support. HZ performed the clinical analysis. SCa and ZB wrote the manuscript. All authors have read and approved the final version of manuscript.

### Conflict of Interest

The authors declare that the research was conducted in the absence of any commercial or financial relationships that could be construed as a potential conflict of interest.
