# Supplementary material for: Glycyrrhizic Acid-Induced Differentiation Repressed Stemness in Hepatocellular Carcinoma by Targeting c-Jun N-Terminal Kinase 1
Source: Front Oncol. 2020 Jan 9;9:1431. doi: 10.3389/fonc.2019.01431 (PMC6962306; doi:10.3389/fonc.2019.01431)
Supplement: Supplementary file 1 [file Data_Sheet_1.docx]

**GA blocked the effect on stemness and differentiation induced by JNK1 over-expression**

To investigate the mechanism of GA in JNK1 over-expression model, we over-expressed JNK1 and performed Western blot assay. The results clearly showed that JNK1 expression was up-regulated in HepG2 and PLC/PRF/5 cells after being transfected with the JNK1 plasmid compared with Vector (Figure S1A). GA alone decreased colony formation, while JNK1 over-expression increased colony formation. Furthermore, GA blocked the promotive effect of JNK1 on colony formation (Figure S1B; one-way ANOVA, ****P* < 0.001). Meanwhile, adding GA alone reduced expression of CSC markers, whereas that of CSC markers was dramatically increased after JNK1 over-expression. In addition, GA inhibited the enhanced effect of JNK1 on stemness (Figure S1C). Similarly, adding GA alone could reverse the poor differentiation of HCC to well differentiation, and the expression of differentiation markers exerted corresponding changes. However, poor differentiation of HCC resulted from JNK1 over-expression could turned to well differentiation after GA added (Figure S1C). These results further demonstrated GA blocked the effect on stemness and differentiation induced by JNK1 over-expression.


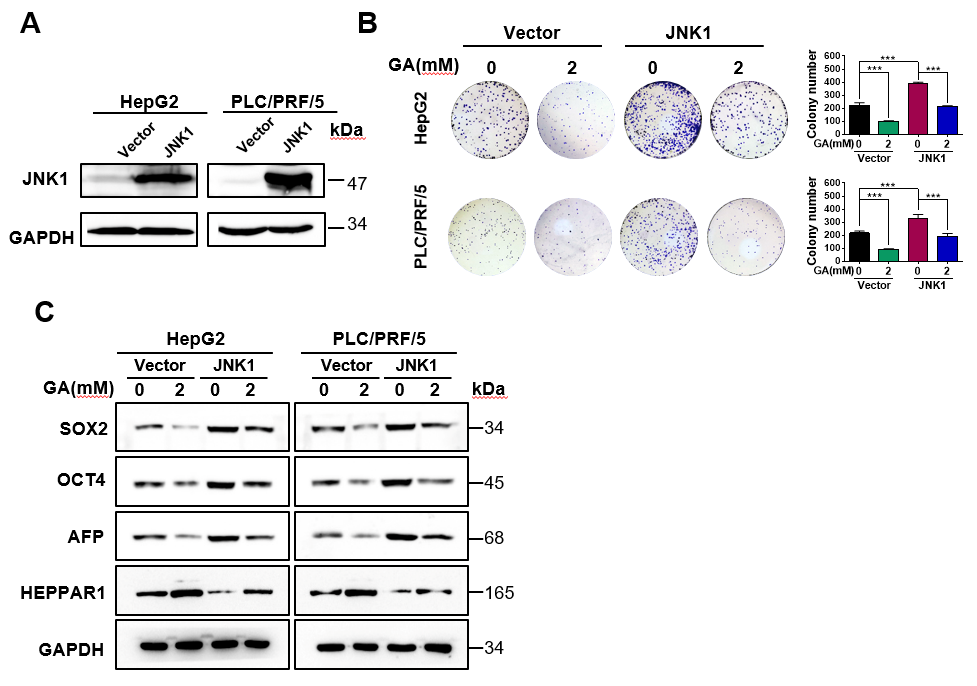


Figure S1. GA blocked the effect on stemness and differentiation induced by JNK1 over-expression.

(A). JNK1 expression in HepG2 and PLC/PRF/5 cells after being transfected with Vector and JNK1 plasmids. (B). Colony formation assay in HepG2 and PLC/PRF/5 cells transfected with Vector and JNK1 plasmids, followed by treatment with DMSO or GA (2 mM) for 48 h. The numbers of colonies were compared (one-way ANOVA; ****P* < 0.001). (C and D). Expressions of representative CSC markers (SOX2 and OCT4) and differentiation markers (AFP and HEPPAR1) were analyzed by Western blot assay.


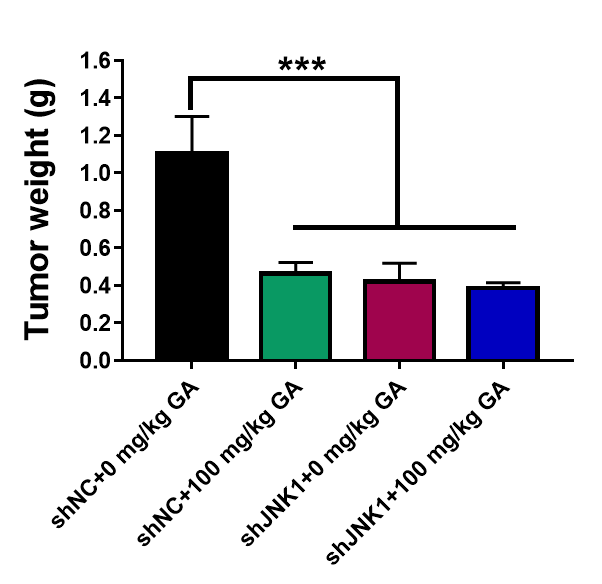


Figure S2. Tumor weight of PLC/PRF/5 transfected with shNC or shJNK1 in BALB/c nu/nu mice treated with 100 mg/kg GA or saline as control. The tumor weight were compared (one-way ANOVA; ****P* < 0.001).
